# Subjective geriatric complaints as predictors of disability and mortality in community-dwelling older adults: a 5-year cohort study

**DOI:** 10.1093/ageing/afaf152

**Published:** 2025-06-05

**Authors:** Hajime Takechi, Akira Tsuzuki, Hiroshi Yoshino, Takenori Okumura, Yoshikiyo Kanada

**Affiliations:** Department of Geriatrics and Cognitive Disorders, Fujita Health University, Toyoake, Aichi 470-1192, Japan; Faculty of Rehabilitation, Fujita Health University, Toyoake, Aichi, Japan; Department of Geriatrics and Cognitive Disorders, Fujita Health University, Toyoake, Aichi 470-1192, Japan; Department of Geriatrics and Cognitive Disorders, Fujita Health University, Toyoake, Aichi 470-1192, Japan; Faculty of Rehabilitation, Fujita Health University, Toyoake, Aichi, Japan

**Keywords:** disability, mortality, quality of life (QOL), subjective geriatric complaints, symptom, older people

## Abstract

**Background:**

Various health-related concerns experienced daily by older adults, designated here as subjective geriatric complaints (SGCs), and are suspected to be early signs of the decline in quality of life (QOL). This study aims to test the hypothesis that SGCs are significant predictors of future disability and mortality among older adults.

**Methods:**

This prospective cohort study was conducted in Japan. A health-related questionnaire was mailed to community-dwelling older adults, and data on the certification of long-term care needs and mortality that occurred over the subsequent 5 years were analysed. The analysis included 10 199 individuals. Thirteen SGCs were classified into six groups. The primary outcome was a composite end point of disability and mortality. Survival time analysis was conducted using Kaplan–Meier analysis and Cox proportional hazard regression models.

**Results:**

The mean age (standard deviation) of participants (52.4% female) at baseline was 73.7 (6.0) years. Over the 5-year study period, 1793 participants (17.6%) were newly certified as requiring long-term care and 931 (9.1%) died. After adjusting for age, sex, depressive mood, and presence of multimorbidity, the hazard ratios (95% confidence intervals) for SGC 1b (circulatory/respiratory complaints) and SGC 3 (neurological complaints) were 1.558 (1.316–1.884, *P* < 0.001) and 1.355 (1.14–1.61, *P* = 0.001), respectively.

**Conclusion:**

These findings suggest that SGCs are independent risk factors for a decline in QOL. Additionally, risk varied across different symptom groups within SGCs. These differences should be carefully considered in the management of health for older adults.

## Key Points

- The identification of symptom groups as independent predictors of poor outcomes in older adults underscores the importance of symptom-focused preventive interventions.
- In this prospective cohort study, we followed community-dwelling older adults without disabilities at baseline to investigate the relationship between factor-clustered subjective geriatric complaints and the incidence of disability and mortality over a 5-year period.
- Our findings further reveal that specific symptom groups, such as circulatory and respiratory complaints, are the strongest predictors of adverse outcomes, followed by neurological and excretory complaints.

## Introduction

In today’s rapidly aging society, many diseases have been overcome owing to advances in medical treatment and health promotion initiatives. However, age-related diseases and conditions are increasing, and the costs associated with the treatment and care of older adults are expected to rise further in the future [1, 2]. Therefore, it is essential to identify factors leading to disability and death at an early stage and take appropriate measures. In addressing the health challenges associated with aging, concepts such as geriatric syndrome, frailty and multimorbidity have been proposed, their relationships with quality of life (QOL) have examined in longitudinal studies, and prevention methods have been explored [3–7].

This study hypothesises various health-related concerns experienced daily by older adults, designated here as subjective geriatric complaints (SGCs), are early signs of the decline in QOL. Similar concepts have been discussed under terms such as self-reported health complaints, subjective health complaints, and self-reported hindering health complaints or symptoms [8–14]. SGCs may exist as health concerns before leading to medical consultation, and it is believed that many older individuals experience health concerns without seeking medical attention [15, 16]. Such health concerns may result in reduced outings and daily activities, potentially increasing the risk of frailty. Consequently, this may lead to a heightened risk of disability or mortality. However, research on SGCs or symptoms in relation to mortality and functional decline remains insufficient [17–21].

In a previous report, the frequency and factor structure of SGCs by age group were presented [22]. While some studies have described the factor structure of symptoms in older populations, longitudinal analyses examining the risk of a decline in QOL based on these factors have not been conducted [9]. Although a few longitudinal studies have reported an association between an increase in the number of symptoms reported by older adults and hospitalisation or mortality, the causal relationship between symptom groups, as analysed by factor structure, and disability or mortality remains unclear [17–21]. Aging induces various changes in organs, but the impact of aging on each organ differs among individuals [23]. Therefore, it is crucial to not only examine the number of symptoms, but also understand the long-term risks associated with the types of symptoms.

Given this background, the present longitudinal study aimed to clarify the significance of SGCs by investigating the relationship between factor-grouped SGCs and the incidence of disability and mortality over a 5-year period.

## Methods

### Study design and participants

This prospective cohort study was conducted using data from a health survey targeting all older citizens of Toyoake city, which has been conducted every 3 years since December 2016. The survey was cross-referenced with the city’s continuously recorded data on long-term care certification and death.

As part of the cohort study, a questionnaire was sent to 14,850 people aged ≥65 years living in the city in December 2016 who had not applied for long-term care certification at the time of the first survey (89.2% of the old adult population in the city). Older community-dwelling adults who had long-term care certification and were thought to have disability at that time were excluded from the study (*n* = 1801, 10.8% of the old adult population in the city). Responses were received from 10 740 people (response rate: 72.4%). After collecting the questionnaires via post, we removed the personal numbers that identified the individuals and created a data set for the analysis. After removing respondents with missing data for the main items, 10 434 people were finally analysed. In the present longitudinal study, 235 people who had relocated to outside the city were excluded, leaving 10 199 participants for the final analysis. This study was approved by the Bioethics Review Committee of the Fujita Health University School of Medicine (HM22–124). Written informed consent was obtained from all participants.

### Procedures and outcomes

The baseline assessment questionnaire included items on age, sex, family composition, body mass index (BMI), multimorbidity and depressive mood. Regarding SGCs, the questionnaire asked, ‘which of the following symptoms may interfere with your daily life?’ and listed 13 symptoms: dizziness, headache, insomnia, urination disorder (pollakiuria and/or urinary incontinence), defecation disorder (constipation and/or diarrhoea), vision impairment, hearing loss, appetite loss, low back pain, arthralgia (including tingling), dysphagia (including choking), shortness of breath and edema. These symptom lists were developed based on existing lists of geriatric syndromes and related publications [7, 22, 24–26]. Symptoms associated with discrete diseases, such as dementia, were excluded from the list. Similarly, conditions typically associated with severe illness, such as delirium or pressure ulcers, which are not common in community-dwelling older adults, were also excluded. Multiple responses for SGCs were allowed, and the number of SGCs per person was designated as the SGC score (0–13). SGCs were classified into following six groups; SGC 1a (excretory complaints; defecation disorder and urination disorder), SGC 1b (circulatory/respiratory complaints; edema and shortness of breath), and SGC 1c (swallowing/sleep complaints; dysphagia, appetite loss, and insomnia). SGC 2 (audiovisual complaints; hearing loss and vision impairment), SGC 3 (neurological complaints; headache and dizziness) and SGC 4 (musculoskeletal complaints; low back pain and arthralgia). The details of classification are described in Appendix 1.

For multimorbidity, the number of the following diseases was counted: hypertension, stroke, heart disease, diabetes, hyperlipidemia, respiratory disease, gastrointestinal disease, renal/urinary tract disease, musculoskeletal disease, trauma, malignant tumour, hematologic and immune disease, ophthalmologic disease and otolaryngologic disease. The total number of diseases was designated as the multimorbidity score (0–14). When indicating the presence or absence of multimorbidity, participants with two or more of these diseases were classified as having multimorbidity [4]. Depressive mood was assessed using two questions about depressive mood and a loss of interest in regular activities. A positive response to either question was considered indicative of depressive mood [27].

The primary outcomes were disability and all-cause mortality. Disability was defined as the first instance of long-term care application and subsequent certification for support or care need after the deadline for the questionnaire responses, which was verified using the records of the city. In Japan’s long-term care insurance system, certification is granted when physical or cognitive decline is observed and care is deemed necessary [28–31]. Therefore, the point at which an individual was certified as requiring long-term care was considered the onset of disability. For all-cause mortality, post-questionnaire deaths were verified using the records of the city. Both were combined as outcomes, with the event date marked as the day of occurrence. Details are described in Appendix 1.

### Statistical analyses

The participants’ characteristics, SGC score, multimorbidity score, and depressive mood status were summarised using basic descriptive statistics, including means, standard deviations (SDs) and proportions as appropriate. Differences between groups were compared using Student’s *t*-test for quantitative variables and the chi-square test for categorical variables.

During the 5-year study period, the occurrence and timing of long-term care certification and death were recorded, the Kaplan–Meier method was used to estimate survival curves, and the log-rank test was applied to compare survival distributions. Cox proportional hazards models were also constructed. Details of models are described in Appendix 1. In the survival analysis, the primary end point was a composite outcome of either long-term care certification or death. When conducting survival analysis by SGC groups, comparisons were made between participants with no SGCs and those with at least one symptom from the analysed SGC groups. All statistical analyses were carried out using IBM SPSS for Windows (ver. 27.0; IBM, Armonk, NY). A two-tailed *P*-value of <0.05 was considered significant.

## Results

The mean age (SD) and BMI of the participants (52.4% female) at baseline were 73.7 (6.0) years and 22.6 (3.1) kg/m^2^, respectively. Regarding SGCs, the average number per person was 1.7 (1.6). The most common SGC was back pain, reported by 32.8% of the participants, followed by visual impairment (27.1%), arthralgia (20.8%), hearing loss (16.1%) and urinary issues (15.4%). The most prevalent conditions for which participants sought medical care or experienced sequelae were hypertension (40.6%), visual impairment (19.6%), hyperlipidemia (14.6%) and diabetes (12.7%) (Table 1). The primary outcomes over the 5-year period were long-term care certification in 1793 participants (17.6%) and death in 931 (9.1%).

**Table 1:** Characteristics of the participants at baseline

Age, years	73.7 (6.0)	
Sex (female n, %)	5349 (52.4)	
BMI, kg/m^2^	22.6 (3.1)	
Depressive mood (present n, %)	3439 (33.7)	
Chronic diseases	n	%
Hypertension	4137	40.6
Ophthalmologic disease	1994	19.6
Hyperlipidemia	1489	14.6
Diabetes	1295	12.7
Heart disease	1010	9.9
Musculoskeletal disease	970	9.5
Renal/urinary tract disease	924	9.1
Gastrointestinal disease	695	6.8
Otolaryngologic disease	609	6
Respiratory disease	471	4.6
Malignant tumour	405	4
Stroke	346	3.4
Trauma	247	2.4
Hematologic and immune disease	147	1.4
Subjective geriatric complaints	n	%
Low back pain	3348	32.8
Vision impairment	2764	27.1
Arthralgia	2118	20.8
Hearing loss	1640	16.1
Urination disorder	1569	15.4
Insomnia	1347	13.2
Defecation disorder	1259	12.3
Dizziness	958	9.4
Shortness of breath	827	8.1
Dysphagia	526	5.2
Headache	512	5
Edema	429	4.2
Appetite loss	213	2.1

Data are expressed as n (%) or mean (SD) unless otherwise specified.

BMI = body mass index. SD = standard deviation.

When comparing participants with no vs. one or more SGCs, the participants with one or more SGCs were significantly older, had a higher number of diseases and SGCs, higher rates of long-term care certification, and higher mortality rates. No significant differences in sex or BMI were observed between groups (Table 2).

**Table 2:** Comparison of participants with and without SGCs

	Any SGC	No SGCs	*P*
	n = 8066	n = 2133	
Characteristics at baseline			
Age, years	74.0 (6.1)	72.5 (5.6)	< 0.001
Sex (female %)	52.2	53.5	0.256
BMI, kg/m^2^	22.6 (3.2)	22.5 (2.9)	0.112
Multimorbidity score	1.6 (1.3)	1.1 (1.0)	< 0.001
SGC score	2.2 (1.5)	0.0 (0.0)	< 0.001
Outcome measure during follow-up			
LTCI certification (%)	19.2	11.6	< 0.001
Death (%)	9.8	6.7	< 0.001

Data are expressed as n (%) or mean (SD), unless otherwise specified.

BMI = body mass index. SGC = subjective geriatric complaint.

LTCI = long-term care insurance.

The presence of at least one SGC was categorised as ‘any SGC’.

**Figure 1:**
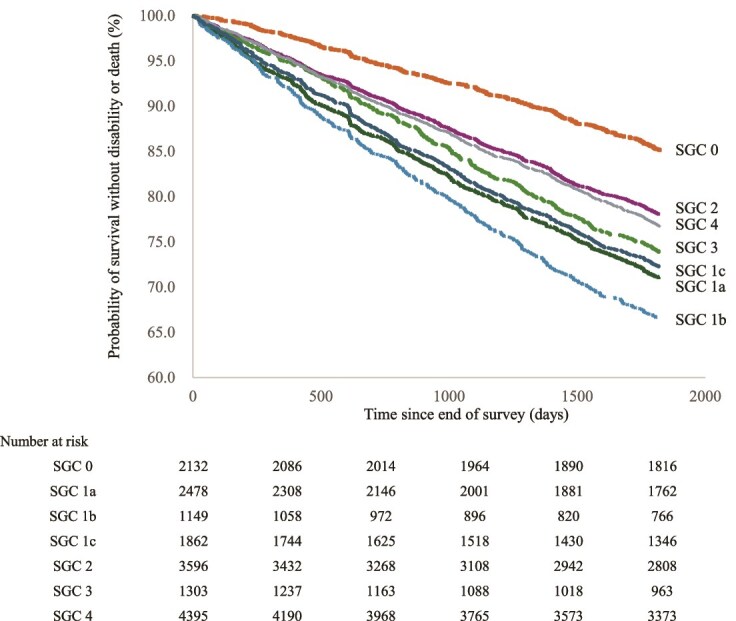
Kaplan–Meier plot showing the probability of survival without disability or mortality according to the different groups of subjective geriatric complaints (SGCs). The y-axis indicates the probability of survival free from disability or death, and the x-axis represents the time (in days) since the end of the survey. Survival curves are shown separately for each SGC group, as labeled in the figure. The curves illustrate the impact of different SGC groups on survival outcomes. Numbers at risk at baseline and at each year from the 1st to the 5th are also shown.

A Kaplan–Meier analysis was conducted using the combined end point of disability or death as the dependent variable, comparing each SGC group (SGC 1a, SGC 1b, SGC 1c, SGC 2, SGC 3, and SGC 4) with the control group (no SGCs). The incidence of the combined end point was significantly higher in all SGC groups (log-rank test, *P* < 0.001 for all comparisons). For instance, the 5-year end point incidence rate was 34% in the SGC 1b group, compared with 15% in the control group. The next highest incidence rates were observed in the SGC 1a, SGC 1c, and SGC 3 groups, respectively (Fig. 1). The median number of SGCs was 1, and the median for the upper half was 2. Therefore, the number of symptoms was divided into four categories: 0 (*n* = 2133), 1 (*n* = 3411), 2 (*n* = 2258) and ≥ 3 more (*n* = 2397). The Kaplan–Meier analysis was performed with the composite end point as the outcome across these four groups, and a significant difference was observed (log-rank test, *P* < 0.001) (Appendix Fig. 1). When mortality alone was analysed as the outcome in the Kaplan–Meier analysis, although the outcome incidence was lower than that for the combined end point, a significant difference was still observed between participants with and without SGCs (Appendix Fig. 2).

Next, a Cox regression analysis was conducted to examine the incidence of the combined end point (Table 3). Because SGCs may be influenced by depressive tendencies and comorbidities, the results were adjusted for age, sex, depressive mood and multimorbidity. Model 1 was adjusted for age and sex only, while Model 2 added depressive mood and Model 3 further adjusted for multimorbidity. In Model 3, the hazard ratios (HRs) with (95% confidence intervals) were as follows: SGC 1b: 1.558 (1.316–1.884, *P* < 0.001); SGC 3: 1.355 (1.14–1.61, *P* = 0.001); and SGC 1a: 1.341 (1.161–1.548, *P* < 0.001). On the other hand, while SGC 2 was significant in Model 1, it was not significant in Models 2 and 3, with HRs of 1.114 (0.973–1.276, *P* = 0.117) and 1.059 (0.923–1.216, *P* = 0.413), respectively.

**Table 3:** Cox regression analysis conducted to examine the incidence of the combined end point

	Model 1				Model 2				Model 3			
	HR	95% CI		*P*	HR	95% CI		*P*	HR	95% CI		*P*
SGC	1.34	1.189	1.51	< 0.001	1.223	1.084	1.381	0.001	1.186	1.05	1.34	0.006
Each SGC												
SGC 1a	1.563	1.366	1.788	< 0.001	1.376	1.196	1.583	< 0.001	1.341	1.161	1.548	< 0.001
SGC 1b	1.862	1.601	2.166	< 0.001	1.643	1.396	1.932	< 0.001	1.558	1.316	1.844	< 0.001
SGC 1c	1.667	1.449	1.919	< 0.001	1.358	1.165	1.582	< 0.001	1.31	1.12	1.532	0.001
SGC 2	1.231	1.08	1.404	0.002	1.114	0.973	1.276	0.117	1.059	0.923	1.216	0.413
SGC 3	1.631	1.399	1.903	< 0.001	1.407	1.188	1.666	< 0.001	1.355	1.14	1.61	0.001
SGC 4	1.369	1.206	1.553	< 0.001	1.248	1.096	1.421	0.001	1.184	1.038	1.351	0.012

Model 1: adjusted for age and sex, Model 2: adjusted for age, sex, and depressive mood, Model 3: adjusted for age, sex, depressive mood, and presence of multimorbidity. SGC = subjective geriatric complaint. HR = hazard ratio. CI = confidence interval.

To explore further the direct relationship between SGCs and multimorbidity, we examined the presence of symptoms in participants with (*n* = 4222) and without (*n* = 5977) multimorbidity. Those with multimorbidity had significantly more SGCs across all categories (for all SGCs; *P* < 0.001), with symptom prevalence being 1.3 to 2.5 times higher compared with those without multimorbidity (Table 4).

**Table 4:** Presence of complaints in participants with and without multimorbidity

		Multimorbidity		
	Total	Absent	Present	*P*
	*n* = 10 199	*n* = 5977	*n* = 4222	
Urination disorder, n (%)	1569 (15.4)	685 (11.5)	884 (20.9)	< 0.001
Defecation disorder, n (%)	1259 (12.3)	576 (9.6)	683 (16.2)	< 0.001
Shortness of breath, n (%)	827 (8.1)	308 (5.2)	519 (12.3)	< 0.001
Edema, n (%)	429 (4.2)	157 (2.6)	272 (6.4)	< 0.001
Dysphagia, n (%)	526 (5.2)	199 (3.3)	327 (7.7)	< 0.001
Appetite loss, n (%)	213 (2.1)	85 (1.4)	128 (3.0)	< 0.001
Insomnia, n (%)	1347 (13.2)	668 (11.2)	679 (16.1)	< 0.001
Vision impairment, n (%)	2764 (27.1)	1423 (23.8)	1341 (31.8)	< 0.001
Hearing loss, n (%)	1640 (16.1)	767 (12.8)	873 (20.7)	< 0.001
Headache, n (%)	512 (5.0)	248 (4.1)	264 (6.3)	< 0.001
Dizziness, n (%)	958 (9.4)	452 (7.6)	506 (12.0)	< 0.001
Low back pain, n (%)	3348 (32.8)	1717 (28.7)	1631 (38.6)	< 0.001
Arthralgia, n (%)	2118 (20.8)	989 (16.5)	1129 (26.7)	< 0.001

The percentage of each complaint was calculated based on the proportion of the presence or absence of each complaint, categorised by the presence or absence of multimorbidity.

## Discussion

This study investigated the relationship between complaints identified through a comprehensive survey of community-dwelling older adults and subsequent 5-year outcomes of disability and mortality. Thirteen common complaints related to health concerns in older adults were grouped into six complaint groups (SGCs) based on factor analysis, and their associations with outcomes were analysed. Among the groups, SGC 1b (respiratory and circulatory complaints) were the most strongly associated with adverse outcomes, followed by SGC 3 (neurological complaints), such as headache and dizziness. While multimorbidity was more prevalent among participants with SGCs, those without multimorbidity still exhibited a high prevalence of SGCs. This finding suggests that many older adults with SGCs do not seek medical attention despite experiencing SGCs. The proper management of SGCs may contribute to early medical interventions or preventive strategies, potentially helping to maintain QOL in older adults.

Although few longitudinal studies have examined the association between symptoms and outcomes such as disability or mortality, Sha *et al*. [19] analysed the relationship between symptoms and hospital admissions or mortality after 1 year. Salanitro *et al*. [18, 20] conducted an 8.5-year longitudinal study on 980 Medicare recipients, analysing hospitalizations and emergency room visits as outcomes, while Sheppard *et al*. [18, 20] studied the same cohort using nursing home admissions as an outcome. Patel *et al*. [17] conducted a 6-year longitudinal analysis of 7609 individuals aged ≥65 years. Although these studies differ in the number and type of symptoms analysed, all reported that a higher number of symptoms was associated with adverse outcomes such as mortality, disability, and hospitalisation [17–20]. The present findings are consistent with those studies. However, we have extended this understanding by showing that specific symptom groups, such as respiratory and excretory complaints, are more predictive of adverse outcomes. In this study, each SGC included two or three symptoms, and the associations with outcomes varied across the six groups, suggesting that identifying specific symptom types, rather than simply counting the number of symptoms, is crucial. It is known both experientially and scientifically that the organs most susceptible to impairment due to aging vary by individual, and thus, future research from this perspective is warranted [23].

Previous longitudinal studies have examined the association between specific symptoms, such as arthralgia and shortness of breath, and reduced QOL [32, 33]. However, few studies have conducted longitudinal analyses of multiple symptoms in the same cohort. Additionally, while some studies have examined the impact of the number of symptoms on outcomes, no longitudinal analyses focussing on which specific symptoms are more likely to be associated with outcomes have been conducted [17–20].

In a previous SGC survey, 82.1% of older adults reported experiencing symptoms that could potentially affect their daily lives [22]. The average number of symptoms per person was 1.72 (*n* = 3286, SD 1.57), and while the number of symptoms increased with age, even among those aged 85–89 years, the average number was 2.29 (*n* = 462, SD 1.89) [22]. As in previous studies, the results of the present analysis suggest that the number of symptoms is strongly associated with outcomes [17–20]. However, it is essential to understand how individual symptoms contribute to these outcomes.

Research on the factor analysis of multimorbidity has shown clear groups of conditions, suggesting that aging is not simply associated with an increase in the number of diseases, but rather, that the types of diseases differ across individuals [34]. Future studies should focus on the relationship between different types of SGCs and multimorbidity, and how these, in turn, relate to outcomes such as disability or death.

In this study, the analysis was restricted to community-dwelling older adults who were not receiving care at the time of the SGC survey. While longitudinal studies have examined symptoms, some have included younger populations or those closer to the end of life [35, 36]. It is important to clarify the target population for risk prediction and define the purpose of symptom assessment as being for early intervention or understanding the current health status in each study. Although the present study focused on individuals aged ≥65 years, some previous studies on symptoms have examined populations aged ≥75 years [8, 10]. Because associations between symptoms and outcomes such as disability or death may differ across age groups, it is necessary to consider age and frailty when conducting preventive interventions. When examining outcomes such as disability or mortality, careful consideration is needed to determine whether to include symptoms directly related to these outcomes [8, 9]. It should also be noted that differentiating physical symptoms from psychiatric disorders such as somatoform disorders can be challenging in older adults, and differences may arise depending on factors such as age group, medical history, and the type of symptoms [37].

In recent years, concepts such as geriatric syndromes, frailty and multimorbidity have been proposed as risk factors for reduced QOL in older adults [3, 4, 7]. Of these, geriatric syndromes encompass a wide range of conditions that arise with aging, leading to blurred boundaries between these conditions [7]. When geriatric syndromes were first proposed, they primarily referred to conditions such as delirium, pressure ulcers, urinary incontinence, falls, and fractures, where aging-related factors interact, thereby necessitating interdisciplinary care [7]. However, symptoms such as palpitations, edema and constipation, as well as phenomena related to multimorbidity, such as polypharmacy, have also been classified as geriatric syndromes [24]. While frailty is often included in the concept of geriatric syndromes, despite the established definition of frailty, there is ongoing debate regarding its diagnostic criteria and the elements that constitute frailty [4]. Moving forward, it will be necessary to deepen our understanding of the interactions between SGCs, frailty, geriatric syndromes, and multimorbidity while advancing proactive interventions to maintain the health and QOL of older adults.

The present study has several strengths and limitations. First, this study was conducted in a single region of Japan. The survey achieved a high response rate, and the end points were accurately recorded. Furthermore, the city represents a mid-sized urban–rural area that reflects an average region of Japan demographically (population density of the city: 2936 persons/km^2^. In Japan, ≥4000 persons/km^2^ is officially defined as a densely inhabited district). However, to generalise these findings, similar studies must be conducted in diverse regions worldwide. Moreover, although the response rate in the present survey was high, it is possible that non-respondents may exhibit different trends. Future studies should aim to clarify the incidence of endpoints of non-respondents. Second, this study used self-reported data to assess SGCs and multimorbidity rather than relying on physician diagnoses or objective measures of symptom severity. Future research should incorporate in-person assessments and medical diagnoses to gain a better understanding of the pathways from SGCs to disability and death. Third, although symptoms were presented as belonging to specific organ systems, in reality, a given symptom may have multiple physiological origins. Therefore, caution is warranted in interpreting symptom groupings as strictly organ-specific.

In conclusion, the present findings suggest that SGCs should be recognised as independent risk factors for QOL decline. In addition, there are differences in the strength of associations between outcomes and specific SGCs, indicating the need for further research on symptom types and their relationship with outcomes.

## Supplementary Material

aa-24-2796-File003_afaf152

## Data Availability

The data supporting the findings of this study are available on request from the corresponding author. The data are not publicly available because of privacy or ethical restrictions.
