# Supplementary material for: Subjective geriatric complaints as predictors of disability and mortality in community-dwelling older adults: a 5-year cohort study
Source: Age Ageing. 2025 Jun 5;54(6):afaf152. doi: 10.1093/ageing/afaf152 (PMC12140101; doi:10.1093/ageing/afaf152)

**Subjective geriatric complaints as predictors of disability and mortality in community-dwelling older adults: a 5-year cohort study**

**Appendices**

Appendix 1. Methods.

Appendix Figure 1. Kaplan–Meier plot showing probability of survival without disability or death based on the numbers of Subjective Geriatric Complaints (SGCs)

Appendix Figure 2. Kaplan–Meier plot showing probability of survival without death based on the different groups of Subjective Geriatric Complaints (SGCs)

**Methods**

**Definition of terms**

In this study, we use the terms “complaint” and “symptom” interchangeably but with specific contextual emphasis. “Complaint” refers to subjective health concerns reported by individuals, often before seeking medical attention, and these form the basis of the data collected in our study. On the other hand, “symptom” is used to describe clinical manifestations that may be more familiar to the medical community. However, even with this distinction in mind, it is challenging to strictly differentiate between these two terms. Therefore, in this paper, both terms are used almost interchangeably.

**Classification of SGCs into 6 groups**

After a preliminary analysis, abdominal pain among initial 14 complaints was excluded from further analysis because it was reported by < 5% of the participants in any age group. In a previous study, 13 SGCs were classified into the following four factors via categorical principal components analysis: SGC 1 (excretory/circulatory/swallowing complaints; defecation disorder, urination disorder, edema, shortness of breath, dysphagia, appetite loss), SGC 2 (audiovisual complaints; hearing loss and vision impairment), SGC 3 (neurological complaints; headache and dizziness), and SGC 4 (musculoskeletal complaints; low back pain and arthralgia). In the present study, based on the previous factor analysis results, we divided SGC 1 into three subcategories considering the number of SGCs per factor, value of factor loading and the similarity of organ systems: SGC 1a (excretory complaints; defecation disorder and urination disorder), SGC 1b (circulatory/respiratory complaints; edema and shortness of breath), and SGC 1c (swallowing/sleep complaints; dysphagia, appetite loss, and insomnia). The significance of keeping the number of complaints consistent as much as possible when comparing subjective complaints from the perspective of specific organ systems is demonstrated in the results of the data analysis. Combined with the other three factors (SGCs 2–4), a total of six SGC groups were analyzed.

**Definition of Outcomes and Rationale for Composite Endpoints**

This study aimed to identify subjective geriatric complaints (SGCs) that could serve as early indicators of future need for long-term care or death among community-dwelling older adults who were not receiving care at baseline (aged ≥65 years, mean age 73.7 years). The study was designed as a five-year longitudinal cohort. Given the relatively good health status of the participants at baseline, the incidence of death during the follow-up period was expected to be relatively low. Therefore, to increase the event rate and capture broader aspects of objective quality of life (QOL) decline, we selected both mortality and long-term care certification as outcomes.

In addition to death, other potential indicators of QOL decline include quantitative measures of physical function, hospitalization, and institutionalization. However, we opted not to use short-term changes such as hospitalization, and considered that a substantial proportion of individuals with decreased QOL may continue to reside at home while receiving long-term care. Thus, we used the certification of need for long-term care as an additional objective outcome, as this is systematically recorded by local authorities, along with mortality.

While long-term care certification is broader and more heterogeneous than death, it is a widely accepted, implemented system in Japan since 2000 and reflects both physical and cognitive impairments. Numerous studies have demonstrated that the level of care certification is associated with QOL (representative references cited in the main text).

In the Japanese long-term care insurance system, older adults are assessed and categorized into eight levels: one for independent status (no need for support), two levels of “Support Required” (Support Levels 1 and 2), and five levels of “Care Required” (Care Levels 1 to 5). In this study, we defined the onset of long-term care need as being newly certified for any of the seven non-independent levels.

In our cohort of 10,199 participants at baseline, 1,793 individuals (17.6%) were newly certified for long-term care (Support Level 1: 288; Support Level 2: 426; Care Level 1: 486; Care Level 2: 282; Care Level 3: 114; Care Level 4: 105; Care Level 5: 92), and 931 individuals (9.1%) died during the five-year follow-up period. We considered these event rates adequate for survival analyses. Although the incidence of long-term care need was nearly double that of death, we did not judge the imbalance to be large enough to preclude their combination as a composite endpoint.

While we considered presenting long-term care need and mortality as separate outcomes, we also recognized that some individuals may experience sudden, severe QOL decline leading directly to death (e.g., from acute myocardial infarction), without first becoming dependent. Thus, using a composite outcome encompassing both events was deemed appropriate for this study. Nonetheless, to address the limitations of composite endpoints, we also present analyses using mortality alone as a supplemental figure.

**Cox Regression Models and Covariates**

Cox regression analysis was adjusted for age and sex, as these factors are known to influence significantly both disability and mortality outcomes in older populations. Further adjustments were made for depressive mood and multimorbidity to account for their potential confounding effects. The reason for using depressive mood as a covariate is that individuals with depressive mood are presumed to report their symptoms more strongly. The reason for using multimorbidity as a covariate is that, when considering the framework of symptoms and diseases, there may be an association between symptom reporting and the number of comorbidities, making it desirable to adjust for this factor.

**Figure legends for appendix figures**

***Appendix Figure 1:*** Kaplan–Meier plot showing the probability of survival without disability or mortality based on the number of subjective geriatric complaints (SGCs). The y-axis shows the probability of survival free from disability or death, and the x-axis represents the time (in days) since the end of the survey. Each line corresponds to a different SGC group: no SGCs (orange), 1 SGC (blue), 2 SGCs (dark blue), and ≥ 3 SGCs (green). The curves demonstrate the impact of the number of SGCs on survival outcomes. Numbers at risk at baseline and the 1st to 5th years are also shown.

***Appendix Figure 2:*** Kaplan–Meier plot showing the probability of survival without mortality based on the different groups of subjective geriatric complaints (SGCs). The y-axis shows the probability of survival free from death, and the x-axis represents the time (in days) since the end of the survey. Each line corresponds to a different SGC group: SGC 0 (orange), SGC 1a (dark green), SGC 1b (blue), SGC 1c (dark blue), SGC 2 (purple), SGC 3 (green), and SGC 4 (gray). The curves demonstrate the impact of different SGC groups on survival outcomes. Numbers at risk at baseline and the 1st to 5th years are also shown.


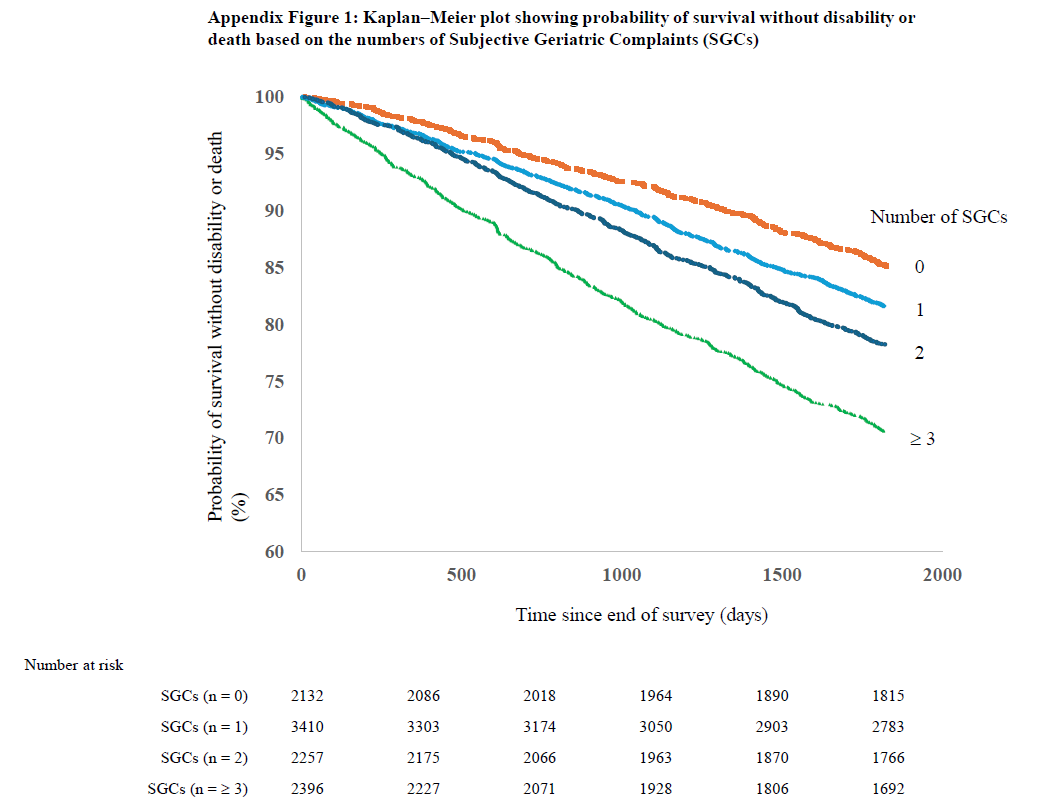


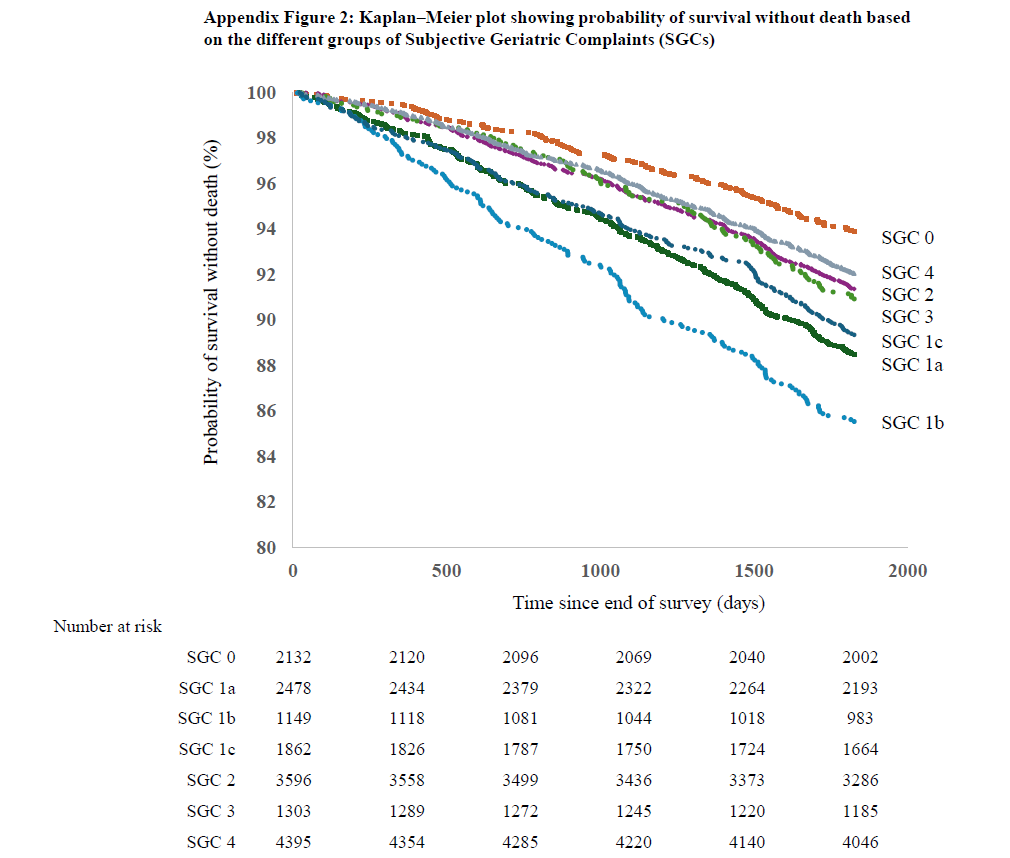

Supplement: aa-24-2796-File003_afaf152 [file aa-24-2796-file003_afaf152.docx]
